# Pre-Rheumatology Referral Consultation and Investigation Pattern in Children with Joint Complaints: Focus on Juvenile Idiopathic Arthritis

**DOI:** 10.3390/children11050600

**Published:** 2024-05-16

**Authors:** Achille Marino, Paola Baldassarre, Cristina Ferrigno, Andrea Biuso, Martina Minutoli, Francesco Baldo, Stefania Costi, Maurizio Virgilio Gattinara, Roberto Felice Caporali, Cecilia Beatrice Chighizola

**Affiliations:** 1 Unit of Pediatric Rheumatology, ASST G. Pini-CTO, 20122 Milan, Italy; francesco.baldo@asst-pini-cto.it (F.B.); stefania.costi@asst-pini-cto.it (S.C.); mauriziovirgilio.gattinara@asst-pini-cto.it (M.V.G.); roberto.caporali@unimi.it (R.F.C.); cecilia.chighizola@unimi.it (C.B.C.); 2 Department of Biomedical and Clinical Sciences, Buzzi Children’s Hospital, University of Milan, 20122 Milan, Italy; paola.baldassarre@unimi.it (P.B.); cristina.ferrigno@unimi.it (C.F.); andrea.biuso@unimi.it (A.B.); martina.minutoli@unimi.it (M.M.); 3 Department of Clinical Sciences and Community Health, Research Center for Pediatric and Adult Rheumatic Diseases (RECAP.RD), University of Milan, 20122 Milan, Italy; 4 Department of Rheumatology and Medical Sciences, ASST G. Pini-CTO, 20122 Milan, Italy

**Keywords:** juvenile idiopathic arthritis, referral, joint complaints

## Abstract

The diagnosis of juvenile idiopathic arthritis (JIA) is often entrusted to the pediatric rheumatologist specialist. Timely referral to a specialized center is crucial. This study aims to assess the consultation and investigation patterns of patients with joint complaints before rheumatology referral. This longitudinal cohort study included patients with joint complaints who were referred to the Pediatric Rheumatology Unit. The cohort included 301 patients (58% female), 50 of them (17%) diagnosed with JIA. Compared to patients with orthopedic conditions or functional diseases, JIA patients had seen more specialists (*p* < 0.01) and received a quicker diagnosis (*p* < 0.01). Patients with early JIA diagnosis (within 3 months from symptoms onset) were younger (8.46 vs. 11.5 years old; *p* = 0.04), more frequently female (78% vs. 47%, *p* = 0.03), and with higher erythrocyte sedimentation rate (ESR) values (37 vs. 9 mm/h; *p* = 0.02) than those diagnosed later. Patients with a late diagnosis of JIA had a significantly longer median time between the first healthcare visit and the PR referral (25 vs. 101 days; *p* < 0.01). The main contributor to diagnostic delay in JIA was the time required for PR referral after the first healthcare consult. Younger age, female sex, and higher ESR values were associated with earlier diagnosis of JIA.

## 1. Introduction

Joint complaints (joint pain, swelling, stiffness) are a frequent symptom in pediatric age, presenting approximately in 10% to 20% of children [1]. It is one of the most common reasons for consulting a pediatrician and a significant cause for referral to a pediatric rheumatologist [2,3], representing a diagnostic challenge in general pediatric outpatient clinics and emergency departments. Musculoskeletal (MSK) disease incidence is rising, and greater attention is required to these conditions. Back in 2003, the World Health Organization had already emphasized the scarce attention given to MSK and rheumatic diseases in pediatric and adult populations [4]. Despite the accrual of evidence about the impact of MSK pain both on patient well-being and on health systems, medical education in this field is still insufficient [5]. The differential diagnoses of MSK pain are multiple, mostly benign, but malignancies and systemic autoimmune diseases need to be considered and promptly excluded [6,7]. Therefore, it is paramount for those involved in children’s care, and especially for the family pediatrician, to be well aware of the relevance of the condition in order to ensure prompt recognition and referral to the appropriate specialist.

### 1.1. Juvenile Idiopathic Arthritis

Juvenile idiopathic arthritis (JIA), the most frequent chronic pediatric rheumatic disease, carries notable pain and disability in children, leading to poor quality of life and an important socioeconomic burden for families and health systems. The prevalence of JIA varies between 16 and 150 per 100,000, while the incidence ranges from 1.6 to 23 cases per 100,000 population, with a pooled annual incidence of 7.8 per 100,000 population [8,9,10]. JIA is not a single entity but refers to any arthritis with no apparent cause, lasting for at least 6 weeks, with onset before age 16 [11]. The term JIA encompasses a heterogeneous group of different diseases classified into clinical categories according to the International League of Associations for Rheumatology (ILAR) classification system: oligoarticular JIA, polyarticular rheumatoid factor (RF) negative JIA, polyarticular RF positive JIA, psoriatic arthritis, enthesitis-related arthritis (ERA), systemic JIA, and undifferentiated JIA [12]. JIA categories vary in terms of disease severity, clinical manifestations, response to treatment, and long-term consequences [13]. JIA is more common in girls than in boys overall (ratio 2:1–3:1) [10]. Clinical presentation is usually dominated by joint swelling and restriction, which are typically more relevant than pain. The joint involvement pattern may be either symmetrical (especially in polyarthritis) or asymmetrical, and particular attention must be paid in the assessment of the temporomandibular joint (TMJ) and sacroiliac joint (SIJ) (a hallmark for ERA), which can both thoroughly be assessed only with MRI [14,15]. Tenosynovitis is often seen on the dorsum of the hands and feet and in the area around the ankles. Around a chronically inflamed joint, local muscle wasting develops rapidly. JIA articular inflammation can result in overgrowth of the bone plates in an evolving skeleton or bone erosion. Abnormal traction, localized growth disturbances, and joint damage can lead to alterations in bone morphogenesis, such as developmental abnormalities of the hip and micrognathia [11].

### 1.2. Benign Joint Hypermobility Syndrome

Benign Joint Hypermobility Syndrome (BJHS) provides an additional condition that should be considered when evaluating a school child who complains of joint disorders. Although sometimes can be asymptomatic, BJHS usually manifests as pain typically affecting the lower limbs, especially the knees [16]. Symptoms often exacerbate in the evening after vigorous activity and may interfere with sleep, allowing a complete recovery by morning. Clinical examination usually reveals joint laxity, as assessed by the Beighton score, without other abnormalities. It is of utmost importance to remember that hyperlaxity may be associated with various genetic conditions, such as Ehlers-Danlos, Marfan, Stickler, Williams syndromes, and trisomy 21. These conditions imply multisystemic involvement, warranting a cardiovascular screening. Physical therapy, emphasizing exercises and activities aimed at improving muscle strength tone and endurance, may provide relief for these patients [17,18,19].

### 1.3. Amplified Musculoskeletal Pain Syndromes

The differential diagnosis of joint complaints in children should also include Amplified Musculoskeletal Pain Syndromes (AMPS). AMPS is a chronic functional disorder with a higher prevalence in adolescent females, characterized by persistent or episodic muscle or joint discomfort whose intensity typically fluctuates. These patients may present allodynia, experiencing pain upon light tactile stimulation, or hyperalgesia, wherein pain responses are exaggerated even to non-noxious stimuli [17,20]. The literature has identified two forms of AMPS: complex regional pain syndrome (CRPS), affecting approximately 1.2 per 100,000 children aged 5 to 15 years [21], and diffuse juvenile fibromyalgia syndrome (JFMS), which accounts for 2% to 6% of cases [22]. Although still unclear, etiopathogenesis has been proposed to involve both central and peripheral nervous system hypersensitivity. Psychosocial factors, such as emotional distress and parental influences, may also impact the onset and progression of these syndromes. Diagnosis typically relies on the exclusion of other organic medical conditions. The management of AMPS requires a multidisciplinary strategy involving a specialized medical team dedicated to patient and family education [17,23,24].

### 1.4. Malignancies

Bone pain may be an early sign of an underlying cancer, preceding blood cell count fall, and it must be promptly recognized to avoid a diagnostic delay that may be prolonged by inappropriate use of steroids [25]. A large, cross-sectional, multicenter study showed that patients with malignant bone tumors had the highest frequency of MSK symptoms, followed by patients with Langerhans histiocytosis, leukemia, sarcomas, and neuroblastoma. In patients with cancer and MSK manifestations, joint pain was the most common symptom, followed by bone pain in the limbs. Few large joints are usually affected, especially the hips and knees. A score to differentiate malignancy-associated joint complaints and JIA has recently been proposed [26].

### 1.5. Septic Arthritis and Osteomyelitis

Infection should be considered as a potential cause of joint complaints. Presentation is usually dramatic: the child is in important discomfort, presenting with fever, severe joint pain and swelling, redness, and limitation of range of motion (ROM). A misleading feature in the younger child is that pain might be referred to sites different from the affected joint, such as the knee for hip arthritis and the abdomen for the sacroiliac joint [27]. Typically, only one joint is affected; however, in immunocompromised children and neonates, more than one joint might be involved. Indolent forms, with normal C Reactive Protein (CRP) and white blood cells (WBC) but typically increased erythrocyte sedimentation rate (ESR) and platelet count, might be seen in younger children, sustained by *Kingella kingae* [28]. Septic bone and joint infections need prompt recognition and antibiotic treatment to prevent systemic dissemination of the infection, potentially progressing up to septic shock, and to avoid cartilage and bone damage, especially when metaphyseal regions and ossification centers are involved [29].

### 1.6. Growing Pains or Childhood Benign Limb Pains

Growing pains, also referred to as childhood benign limb pain, provide a frequent reason to complain to the pediatrician, affecting up to 49% of children aged 3 to 12 years [30]. The first description of this condition dates back to 1986, to be later refined by Evans [30,31]. These pains typically manifest as recurrent episodes of mild to moderate discomfort, usually bilateral, often occurring in the evening or at night. Generally considered benign and self-limiting in otherwise healthy children. The diagnosis of growing pains mainly relies on clinical manifestations; blood tests and imaging studies are usually unnecessary. Management typically entails massage, warmth, and occasional pain relievers. While growing pains typically subside with growth, any modifications in pain characteristics or the onset of new signs or symptoms require further evaluation [17,32,33].

### 1.7. Reactive Arthritis and Transient Synovitis of the Hip

The term reactive arthritis includes a group of several inflammatory joint disorders in which an extra-articular infection precedes the development of joint manifestations [34]. Articular inflammation is caused by an infectious agent that, unlike what happens in septic arthritis, does not directly invade the joint. The main sites of infection are in the intestine, respiratory system, and, in adolescents, the genitourinary tract. The most common intestinal infections are those caused by Shigella, Salmonella, Yersinia enterocolitis, pseudotuberculosis, and Campylobacter jejunii. Nevertheless, reactive arthritis might be caused by extraarticular viral infections (e.g., Rubella, Parvovirus B19, CMV, EBV, HBV, VZV), especially in children [35]. A particular and relatively common condition in pediatric age is the transient synovitis of the hip. This inflammatory process affects the hip, with acute onset and rapid evolution. Pain is often referred to the thigh down to the knee; fever and inflammation markers are mostly absent, while ultrasound shows joint effusion with mild synovial thickening. Hip movements are limited, and walking is either impossible or difficult, with a limp. The acute onset, age (3–10 years), specific localization, and rapid recovery (<2 weeks) are rather characteristic elements of this condition [36]. Non-steroidal anti-inflammatory drugs (NSAIDs) are usually sufficient to resolve painful symptoms and joint swelling [37].

### 1.8. Assessment of Joint Complaints and Diagnostic Tests

An optimal approach to joint complaints in children relies heavily on accurate history-taking and a thorough joint examination in order to identify the best diagnostic pathway. A detailed medical question covering family history and past medical history with special attention to potential precipitating factors (e.g., trauma or infection), and accompanying signs/symptoms (e.g., rash, fever, gastrointestinal manifestations) should also include a comprehensive description of the pain, considering parameters such as intensity, location, onset, triggers, duration, quality, potential temporal or seasonal variations, pain relief measures, aggravating and alleviating factors, interference with the child’s physical, psychological, and social functioning, as well as its impact on daily life [7,17,32,38]. Indeed, mechanical joint pain is often acute in onset and punctiform and exacerbates at the end of the day or with overuse (Table 1). Conversely, inflammatory pain tends to be described as a dull aching pain or simply as stiffness, worsening in the morning and improving with activity [39]. The physical examination should start with the observation of the child’s spontaneous motor behavior and should be conducted with the child undressed. A useful and feasible tool for assessing the musculoskeletal system in school-aged children is pGALS (pediatric Gait, Arms, Legs, Spine), designed for use by non-specialist healthcare practitioners [40].

The hallmarks of inflammatory arthritis at examination encompass joint swelling, reduced range of motion, and tenderness. Gait assessment must be conducted considering the child’s age, and tiptoe and heel walking should be evaluated, as they are valuable tools for assessing foot and ankle involvement [6,40].

Investigations may be warranted in cases of severe or persistent pain or if accompanied by systemic symptoms such as fever, rash, or swelling, including blood tests, X-rays, and ultrasound scans. Blood count and inflammatory markers ESR and CRP are informative in the diagnostic process for joint pain. However, they may be normal or falsely negative [11,32,41]. Lactate dehydrogenase (LDH) may be important in discriminating malignancy from JIA, and a combination of increased values of LDH with low neutrophils and low hemoglobin is a red flag, necessitating further investigations [42]. Second-level laboratory tests, such as an autoantibody profile including antinuclear antibodies (ANA), rheumatoid factor (RF), anti-citrullinated protein antibodies (ACPA), and anti-double stranded DNA (dsDNA) antibodies, are not helpful as initial screening tools. Instead, they are useful in the subcategorization of JIA or in case another systemic autoimmune disease is suspected [43]. Among imaging studies, X-rays represent the initial investigation in subjects with joint pain thanks to the accessibility and ability to detect bone abnormalities such as fractures, dislocations, or bone deformities. Joint ultrasound is a non-invasive, real-time operator-dependent imaging technique, which should be performed by experienced personnel in the evaluation of conditions like tendonitis, bursitis, or soft tissue masses. Due to its high sensitivity for detecting joint effusion, joint ultrasound can be a first-line tool to diagnose or exclude arthritis [44]. Computed tomography (CT) and magnetic resonance imaging (MRI) scans are second-line investigations requested to complete the diagnostic process and better characterize MSK lesions. However, MRI is the gold standard for assessing inflammatory involvement of sacroiliac joints, temporomandibular joints, and the spine [7,32], while CT is rarely employed in the diagnostic pathway of JIA.

### 1.9. Aim of the Study

Since the concept of a “window of opportunity” exists to modify the natural history of JIA and, therefore, the patient prognosis has become more and more accepted, an early referral to PR centers and subsequent prompt initiation of appropriate therapy has become crucial [45,46,47,48]. However, studies from all over the world have shown that, despite the heterogeneity in the modalities to access healthcare, children with JIA are referred to PR centers with a significant delay [20,49,50,51,52,53,54,55,56,57,58,59,60,61,62,63,64,65,66]. The reasons are related to the natural course of the disease itself, which has a remitting behavior and often presents insidiously with painless subclinical swelling. Furthermore, the child may be initially referred to a specialist other than a pediatric rheumatologist. The disease is usually not diagnosed after the first PR consultation, and the time until the final diagnosis can be long and involve unnecessary procedures [58]. This study aims to assess the consultation and investigation patterns that patients with joint complaints underwent before rheumatology referral. For patients that were ultimately classified as JIA, we also sought to identify items in the pre-rheumatology referral consultation and investigation patterns that could predict an early diagnosis.

## 2. Materials and Methods

This longitudinal cohort study included consecutive patients with joint complaints who were referred to the Pediatric Rheumatology Unit of our hospital for the first time between 1 April 2022 and 31 March 2024. Data were recorded retrospectively until 31 May 2023; afterward, data were collected prospectively. Joint complaints were defined as the presence of joint pain, swelling, stiffness, or a combination thereof. The following details were recorded: demographical data, the date of symptoms’ onset, the date of the first healthcare consult, the type and number of healthcare practitioners consulted, the specialist who decided the PR referral, whether the patient had an ophthalmological evaluation before PR referral, blood tests, in particular ESR, antistreptolysin O titer (ASO), ANA, RF, and ACPA. The following radiological investigations performed before the PR referral were also collected: X-rays, joint ultrasound, and MRI. For each patient, the definitive diagnosis and its date were also collected.

Patients were diagnosed with JIA according to the ILAR classification criteria [12]. Otherwise, according to the attending physician, subjects were classified as having an orthopedic/mechanical problem or functional (and then labeled under the term AMPS) or other diseases. Among mechanical/orthopedic disorders, the most frequent were benign Joint Hypermobility Syndrome, osteochondritis, patellofemoral pain syndrome, osteochondrosis such as Legg-Calvé-Perthes disease, Osgood-Schlatter disease, Scheuermann disease. The Adherence to the Health Insurance Portability and Accountability Act of 1996 and the principles outlined in the Declaration of Helsinki were ensured. Approval was obtained from our center’s institutional review board (IRB) (4085_S_P). Patient inclusion required informed consent from patients or legal guardians for medical chart data collection.

### Statistical Analysis

Continuous variables were described using median, minimum, maximum, or interquartile range (IQR), while categorical variables were presented as percentages. The normality of variable distribution was assessed using the Shapiro-Wilk test. Variations in continuous variables were analyzed using the Mann-Whitney non-parametric test or the Kruskal-Wallis test for more than two independent groups. Relationships between categorical variables were examined using either the Chi-Square test or Fisher’s exact test, as appropriate. A significance level of *p* ≤ 0.05 was applied. The analyses were conducted using R Studio (version RStudio 2021.09.2+382 for macOS).

## 3. Results

The cohort included 301 patients (58% female) who presented at our institution for joint complaints during the study period (Table 2). The most common reason for patient referral was pain localized in the joints of lower limbs (176 subjects, 58.5%).

About one-third of the cohort (97 out of 301 subjects) had a rheumatological diagnosis (97/301), as shown in Table 2. The most common inflammatory condition was JIA, accounting for more than half of the rheumatological diagnoses. This was followed by reactive arthritis and transient synovitis of the hip, which together accounted for one-third of the diagnoses. It is worth noting that one patient initially classified as having chronic non-bacterial osteomyelitis (CNO) also had significant acne and was later diagnosed with synovitis, acne, pustulosis, hyperostosis, and osteitis (SAPHO). Other diagnoses included (1 case each) systemic lupus erythematosus (SLE), undifferentiated connective tissue disease (UCTD), pachydermodactily, an isolated Raynaud phenomenon, bilateral Baker cysts (1 case), and idiopathic uveitis.

Beyond inflammatory diseases, we assessed three patients who received a diagnosis of tumor: one benign (pigmented villonodular synovitis of the knee) and two malignant (Ewing sarcoma and neuroblastoma). Furthermore, two patients with unilateral knee swelling were eventually diagnosed with arteriovenous malformations (AVMs).

### 3.1. Pre-Rheumatology Referral Consultation and Investigation Patterns in Patients with JIA, Orthopedic Disorders and AMPS

We then tried to elucidate the consultation and investigation patterns that patients with joint complaints underwent before being referred to the PR, comparing patients subgrouped upon the final diagnosis: those ultimately diagnosed as JIA, those with an orthopedic disorder, and those classified as having AMPS. Patients with neoplasia, rheumatological conditions other than JIA, and those with other or missing diagnoses were excluded from the analysis.

In our cohort, JIA patients had seen more specialists and received a quicker diagnosis compared to the patients with other diagnoses (Table 3). On the other hand, AMPS patients were older and underwent fewer radiologic investigations before the PR referral (Table 3). Notably, a statistically significant difference in ESR values emerged across the three subgroups of patients (*p* < 0.01) (Table 3). Sex distribution and family history for autoimmune disease were similarly distributed in the three subgroups, with female sex being predominant in all three groups. The three groups were also comparable in terms of timing for consultation (time from first symptom onset to the first healthcare consultation, time from the first healthcare consultation to the PR referral), even though the time to first PR consultation was lower for JIA patients. Most patients presented blood test results at PR consultations; no significant difference in blood test requests emerged in patients subclassified upon the final diagnosis (ASO titer, RF, and ANA were available for approximately half of the patients in each diagnostic group) (Table 3).

### 3.2. JIA Patients and Timing of Diagnosis

The JIA subgroup included 32 oligoarticular JIA, 11 polyarticular RF negative JIA, and 7 ERA.

Oligoarticular JIA patients had the lowest time from symptoms onset to the first healthcare consult (8 days, range 0–61; *p* = 0.01), while ERA patients exhibited the longest time from the first healthcare consultation to the PR referral (median 385 days, range 12–708; *p* = 0.08) and to the final diagnosis (median 456 days, range 102–1096; *p* = 0.01). There were no differences among JIA subtypes regarding the number of doctors seen or the number of radiologic investigations conducted before the PR referral.

The median number of doctors consulted before PR referral was 2 (range 1–4). The general pediatrician was consulted in 36 cases, mainly as the first healthcare consult (32/36 cases). Half of the JIA cohort encountered an orthopedic surgeon, mainly as the second healthcare practitioner (14/25 cases). The emergency room doctor saw 24 patients, mostly as the first (11 times) or the second (11 times) healthcare practitioner. The referral to PR has been made mainly by the general pediatrician (21 times) and the orthopedic surgeon (15 times). To note, 4 patients, all with JIA-associated uveitis, were referred by the ophthalmologist. Overall, 11/50 patients (22%) had an ophthalmological evaluation with uveitis screening before the PR referral. The median number of radiological investigations was 1, ranging from 1 to 3. X-rays were performed on 16 patients, articular ultrasounds on 22, and MRIs on 18.

To investigate pre-referral factors associated with an early diagnosis, patients with JIA were categorized based on the timing of the final diagnosis: those diagnosed within 3 months from symptom onset (Early diagnosis) and those diagnosed after 3 months from the first clinical manifestation (Late diagnosis) (Table 4).

The age at the first PR visit was significantly lower in the early diagnosis group compared to the late diagnosis group (*p* = 0.04). Conversely, the rate of boys was considerably higher among patients with late diagnosis (*p* = 0.03). Patients with an early diagnosis had higher ESR values (*p* = 0.02). It is worth noting that patients with a late diagnosis had a significantly longer time between the first healthcare visit and the PR referral (*p* < 0.01), while there was no significant difference between the early and late diagnosis groups in terms of the time from symptom onset to the first healthcare consultation (Table 4). On the other hand, the number of doctors encountered and the number of radiologic investigations conducted before the PR referral were similar between the two groups.

## 4. Discussion

**Figure 1:**
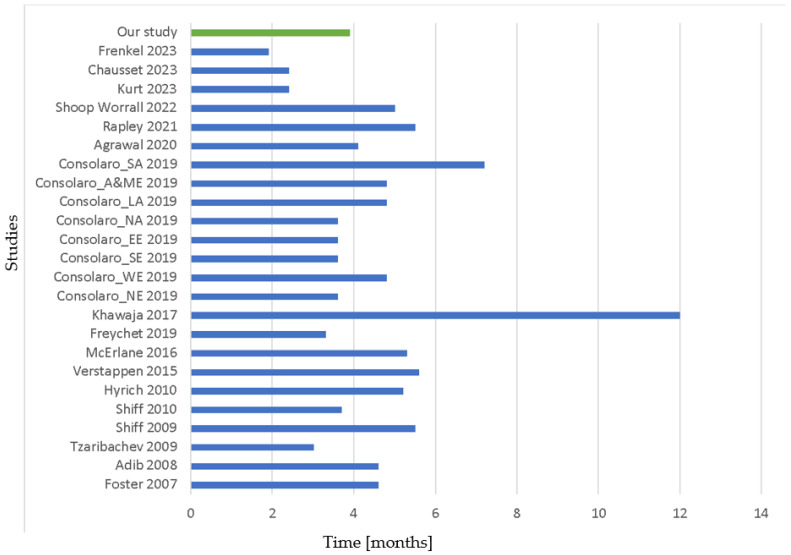
Time to diagnosis or referral to pediatric rheumatology center. Time is expressed in months. SA: Southeast Asia; A&ME: Africa and Middle East; LA: Latin America; NA: North America; EE: Eastern Europe; SE: South Europe; WE: Western Europe; NE: Northern Europe. Studies are reported as first author and year of publication [49,50,51,52,53,54,55,56,57,58,59,60,62,63,64,65,66].

The present is the first study to compare the pre-rheumatology referral consultation and investigation patterns reserved for patients ultimately classified as JIA as compared to subjects diagnosed with mechanical and functional disorders. Over a 2-year timeframe, 17% of the subjects referred to our institution for joint complaints were ultimately diagnosed with JIA. Patients with JIA received the final diagnosis earlier compared to subjects with mechanical or functional disorders, at a median time of 3.9 months. This figure is consistent with previous studies, where the time from the onset of symptoms to JIA diagnosis ranged from 1.9 to 12 months, with a median of 4.6 months (Figure 1) [49,50,51,52,53,54,55,56,57,58,59,60,62,63,64,65,66].

**Figure 2:**
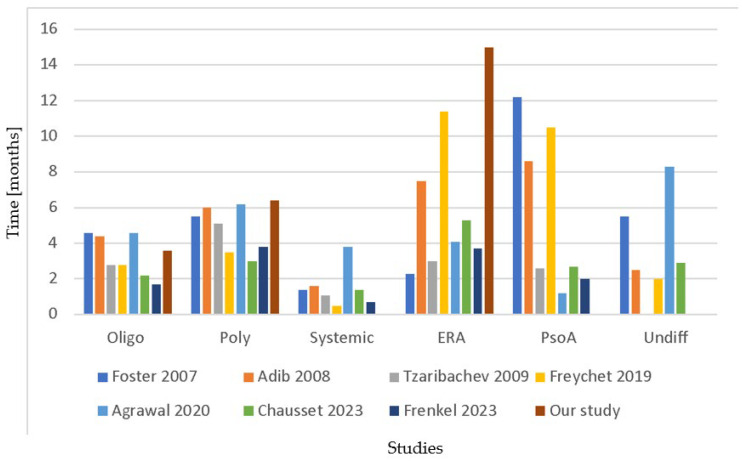
Time to diagnosis or referral to pediatric rheumatology center stratified for JIA categories. Time is expressed in months. Oligo: oligoarticular JIA; Poly: polyarticular JIA; Systemic: systemic JIA; ERA: enthesithis-related arthritis; PsoA: psoriatic arthritis; Undiff: undifferentiated JIA. Studies are reported as first author and year of publication [49,50,51,58,60,65,66].

To note, in our cohort, time to diagnosis varied significantly among JIA categories, with ERA patients obtaining a diagnosis after a median of 15 months, a significantly longer time than subjects with oligoarticular and polyarticular JIA. Consistently, the few available studies analyzing differences among JIA subtypes evinced that ERA, together with psoriatic arthritis, is usually diagnosed later while oligoarticular JIA is typically recognized earlier (Figure 2) [49,50,51,58,60,65,66].

Before PR referral, JIA patients encountered more doctors than subjects with other diagnoses, but there was no firm evidence that they had been investigated with more radiological investigations and blood tests conducted than patients with different diagnoses. ANA was performed in about half of the patients with JIA, a rate similar to those with orthopedic and functional disorders. Interestingly, despite the lack of indication, the ASO titer was performed in a non-negligible percentage of patients across the three groups (18–36%).

In our cohort, an early JIA diagnosis, formulated within 3 months of symptom onset, was significantly influenced by the time span between the first healthcare consultation and the PR referral (Table 4). Additionally, younger age, female sex, and higher ESR values were associated with a faster JIA diagnosis. It was observed that ESR values were higher in the JIA group as compared to patients having other diagnoses, as shown in Table 3. It is worth noting that around 16% of the subjects who were eventually diagnosed with JIA did not undergo laboratory tests before the PR referral. Therefore, it is recommended that an ESR test be conducted as soon as possible in cases where arthritis is suspected.

The role of age at presentation in driving an early diagnosis of JIA has emerged in different cohorts. In a large UK cohort, younger age was found to be a significant predictor of early diagnosis. Children under 5 years old were diagnosed with JIA earlier than older children, and the time to diagnosis increased with age [63]. An Israeli research group reported that younger age and female sex were linked to a quicker diagnosis. A Turkish study with 198 JIA participants found a link between delayed PR referral, older age, and low ESR. They also noted an association with low back pain and enthesitis [64].

Interestingly, in our cohort, few patients had their eyes checked before being referred to PR (22%). Such a figure aligns with the literature where this rate varies between 0–21% [49,52,57]. In a 2020 systematic review, older age at presentation was associated with diagnostic delay. Furthermore, despite including studies from several countries with different healthcare systems, the time to PR referral was relatively consistent across different countries [67].

The current study is flawed by several limitations that should be acknowledged, as the partial retrospective design and the monocentric nature, which could prevent the extrapolation to the whole national JIA population. Parents’ socioeconomic status and educational levels were not collected.

## 5. Conclusions

In our cohort, the main contributor to diagnostic delay in JIA was the time required for PR referral after the first healthcare consult. Younger age, female sex, and higher ESR values were associated with earlier diagnosis of JIA. It would be advisable to establish referral criteria in order to facilitate prompt access to a PR center and to avoid unnecessary investigations.

## Figures and Tables

**Table 1:** The main features of inflammatory and noninflammatory pain in children.

Inflammatory and Noninflammatory Pain in Children		
	Inflammatory	Non-Inflammatory
**Worsening in the morning, after night rest**	+++	−
**Worsening after exercises**	−	variable
**Morning stiffness**	+++	−
**Pain characteristics**	within the affected joint but without precise site	can be punctual, well-defined or diffuse
**Intensity**	variable	variable
**Response to NSAIDS**	partial/good	variable
**ROM restriction**	can be restricted	usually not restricted

+: Presence, higher presence: higher numbers of +, −: absence.

**Table 2:** The general features of the cohort.

	Overall (N = 301)
**Sex, female**	174 (57.8%)
**Age at the first PR consult, years, median [IQR]**	9.97 [7.07–12.67]
**Time latency between symptoms onset and the first healthcare consult, days, median [IQR]**	14.0 [0–123]
**Time latency between the first healthcare consult and the PR referral, days, median [IQR]**	57.0 [27–237]
**N of healthcare provider consulted before the PR referral, days, median [Min, Max]**	1.00 [1, 5.00]
**Diagnosis**	
*Rheumatological diagnosis*	
JIA	50 (16.6%)
Reactive Arthritis	21 (7%)
Transient synovitis of the hip	14 (4.6%)
CNO	4 (1.3%)
JDM	2 (0.7%)
Other rheumatological diagnosis	6 (2%)
*Non-rheumatological diagnosis*	
Orthopedic cause	147 (48.9%)
AMPS	25 (8.3%)
Neoplasia	3 (1%)
Other diagnosis	16 (5.3%)
Missing	13 (4.3%)

IQR: interquartile range; JIA: juvenile idiopathic arthritis; CNO: chronic non-bacterial osteomyelitis [one of CNO patients had SAPHO, see the text]; JDM: juvenile dermatomyositis. AMPS: Amplified Musculoskeletal Pain Syndromes.

**Table 3:** Comparison among juvenile idiopathic arthritis (JIA), orthopedic conditions, and Amplified Musculoskeletal Pain Syndromes (AMPS).

	JIA (N = 50)	Ortho (N = 147)	AMPS (N = 25)	*p*
**Age at the first PR visit, y**				
Median [IQR]	10.1 [4.72–13.92]	9.97 [7.55–9.97]	11.43 [10.13–14.09]	0.03 *
**Sex**				
Female	29 (58.0%)	86 (58.5%)	14 (56.0%)	0.98
Male	21 (42.0%)	61 (41.5%)	11 (44.0%)	
**Positive Familial History for autoimmunity**				
No	32 (64.0%)	79 (53.7%)	15 (60.0%)	0.11
Yes	18 (36.0%)	49 (33.3%)	8 (32.0%)	
**N of specialists before PR referral**				
Median [Min, Max]	2.00 [1.00, 4.00]	1.00 [0, 5.00]	1.00 [1.00, 4.00]	<0.01 *
**Time from symptoms onset and first healthcare consult, days**				
Median [IQR]	14.0 [3–43.25]	30.0 [0–324.2]	14.0 [5.5–63]	0.26
**Time from first healthcare consult and the PR referral, days**				
Median [IQR]	56.0 [19.25–154.25]	83.0 [33–307]	101 [41.5–190]	0.27
**Blood Test before PR referral**				
Not conducted	8 (16.0%)	39 (26.5%)	6 (24.0%)	0.32
Conducted	42 (84.0%)	108 (73.5%)	19 (76.0%)	
**ASO**				
Not conducted	37 (74.0%)	99 (67.3%)	16 (64.0%)	0.18
Conducted	9 (18.0%)	43 (29.3%)	9 (36.0%)	
**ANA**				
Not conducted	23 (46.0%)	70 (47.6%)	12 (48.0%)	0.48
Conducted	25 (50.0%)	73 (49.7%)	13 (52.0%)	
**RF**				
Not conducted	26 (52.0%)	90 (61.2%)	15 (60.0%)	0.36
Conducted	21 (42.0%)	53 (36.1%)	10 (40.0%)	
**N of radiologic investigations before PR referral**				
Median [Min, Max]	1.00 [0, 3.00]	1.00 [0, 3.00]	0 [0, 3.00]	0.02 *
**Time from symptom onset to definitive diagnosis, years**				
Median [IQR]	0.32 [0.17–0.69]	0.88 [0.29–2.13]	0.82 [0.37–1.12]	<0.01 *
**ESR value, mm/h**				
Median [IQR]	20.5 [8.25–50.75]	8.00 [5–16.25]	11.5 [5.5–25.5]	<0.01 *

IQR: interquartile range; PR: pediatric rheumatologist; N: number; ASO: antistreptolysin O titer; ANA: antinuclear antibody; RF: rheumatoid factor, ESR: erythrocyte sedimentation rate. * Statistically significant.

**Table 4:** Early (within 3 months from symptoms onset) vs. Late diagnosis of JIA.

	Early Diagnosis (N = 18)	Late Diagnosis (N = 32)	*p*
**Age at the first PR visit, y**			
Median [IQR]	8.46 [2.95–10.16]	11.5 [6.82–14.61]	0.04 *
**Sex**			
Female	14 (77.8%)	15 (46.9%)	0.03 *
Male	4 (22.2%)	17 (53.1%)	
**Time from symptoms onset and first healthcare consult, days**			
Median [IQR]	7.50 [3.5–23.25]	14.0 [0–80.25]	0.24
**Time from first healthcare consult and the PR referral**			
Median [IQR]	25.0 [10.25–36.75]	101 [62.75–388.5]	<0.01 *
**N of specialists before PR referral**			
Median [Min, Max]	2.00 [1.00, 3.00]	2.00 [1.00, 4.00]	0.35
**N of radiologic investigations before PR referral**			
Median [Min, Max]	1.00 [0, 3.00]	1.00 [0, 3.00]	0.20
**ESR value, mm/h**			
Median [IQR]	37.0 [20.75–58.25]	9.00 [7.25–36.5]	0.02 *

IQR: interquartile range; PR: pediatric rheumatologist; N: number; ESR: erythrocyte sedimentation rate. * Statistically significant.

## Data Availability

The data that support the findings of this study are available from the corresponding author, A.M., upon reasonable request. Data are not publicly available due to ethical reason.
